# Supplementary material for: Impact of chart image characteristics on stock price prediction with a convolutional neural network
Source: PLoS One. 2021 Jun 23;16(6):e0253121. doi: 10.1371/journal.pone.0253121 (PMC8221485; doi:10.1371/journal.pone.0253121)
Supplement: Attachment — Submitted filename: Revision Summary2021.01.12.docx [file pone.0253121.s001.docx]

**Revision Summary**

ID: PONE-D-20-29152.R1

Paper Title: Impact of Chart Image Characteristics on Stock Price Prediction with a Convolutional Neural Network

Reviewer 1

| Comments | Response |
| --- | --- |
| I cannot see any novelty in this work. There are many publications in this regard except the dataset used. | As you pointed out, the study of predicting stock prices with images has begun. Many example studies have been cited in the paper. However, there is still no research on ‘which’ characteristics of a chart image significantly affect the performance of deep learning-based stock price predictions. This is the novelty of this paper. This paper has been researched from this angle and its features are presented in the conclusion section. |
| Since dropout and filter-size are obvious parameters that affect CNN learning- so hypothesis 2 needs reformulation (hypothesis formulation adds no more value to this work). | As the reviewer mentioned, filter size and dropout are factors that influence the CNN model. In addition, in this paper, a hypothesis was created based on the idea that there will be a difference in the degree to which filter size and dropout affect each image feature. The result of Hypothesis 2 and 3 means that when selecting the characteristics of the stock chart, the filter parameter and dropout must also be changed according to the image characteristics. Therefore, the authors treated these parameters as mediating rather than independent variables. |
| There is no cohesion between the research hypothesis and conclusion. | The authors modified the conclusion as advised, adding information about the hypotheses to the conclusion. |
| Stock market prediction is a random walk and includes non-linear dynamics, authors are unable to address these issues in their experiments as well as conclusions. | Although stock market prediction appears to be a random walk, a stock price expert looks at a company's stock price chart and predicts whether a company's stock price will rise or fall based on his knowledge and experience. The results of the performance test in this paper can be evaluated as showing the possibility that CNN can acquire knowledge to some extent imitate the implicit knowledge of stock price prediction experts using charts.  This paper assumes that people who predict stock prices with only charts without numerical data on stock prices have image-based forecasting knowledge. In this study, the authors tried to augment their predictive ability through deep learning. It would be appreciated if you could understand the significance of this study in this way.  These are newly added in conclusion. |
| Authors need to shed light on how chart-based stock prediction is feasible over technical indicator-based prediction. | Table 8 has been revised to show that using chart images results in more accurate stock price prediction than numeric and text-based prediction.  The authors are considering a method of combining image-based prediction and technical indicator-based prediction for future research. Information to this effect has been provided in the discussion section of the revised version. |
| The Paper lacks the details about image feature representation by CNN. | The process of creating an image is as follows.  1. Daily data for 5 years (2015-2019) was collected on 789 companies included in the KOSPI Index calculation in the Dataguide program.  2. Images were composed of data collected on a monthly basis.  3. According to the image characteristics shown in Table 2, 30 images of various types were created for each month with RStudio software.  4. After the images were created, a label for each was automatically generated with the name of the image, which was then compared with the closing stock price 5 days later. Please see the following example.    5. Finally, if the image name rate of return was greater than 0, 1 was automatically added to the end of the label; otherwise, 0 was automatically added.  This text has been added to section 4.1. |
| The information provided about image characteristic is ambiguous. | The information has been clarified in Table 2 and image characteristics have been explained better in the text. In the experiment for this paper, deep learning was performed by generating various chart images according to the characteristics listed in Table 2. |
| Model validation is weak. The author needs to specify the size of the increase dataset, decrease dataset, and Size of training, validation, and testing dataset explicitly. | In order to obtain a set of images for learning, a dataset was created with daily data for all companies listed on the KOSPI Index, and a stock price chart for each image characteristic was created. Total amount of data: 12 (months)*5 (years)*789 (number of companies)*30 (chart image types). Then, 90% of the training set and 10% of the test set were selected randomly so learning and inference could be performed.  This information has been added in the revised manuscript. |
| Detail of CNN network training parameters is missing such as loss function. | We added the following information about parameters in the revised manuscript (p. 14).  Parameters:  Loss function : RMSE  Activation function: {ReLU, softmax}  Image size = 28 * 28 |
| Since the proposed CNN has very limited layers, the network overfitting might be the issue, the author needs to verify this with network generalization capability. | The stock price images created for this study were relatively simple. In our experience, the more layers we set, the worse the performance, and the more parameters we included, the greater the number of calculations. Therefore, we concluded that there was no issue of overfitting. |
| The comparison of the presented result with the previous result is not comprehensive. The reference cited in Table 7 (Namely Gunduz [et.al](http://et.al/) -2017, Nelson et.al-2017) are missing in the reference list. | Thank you. The reference is newly added. |
| The validity of result presented in the paper need to be tested on benchmark datasets as author proposed their own CNN, instead of standard CNN such as VGG, and AlexNet, there are many CNN parameters still need to be optimized in the network such as numbers of layers, optimizers, activation functions, and normalizations. | The authors optimized the network as follows:  Parameter: {Values}  Filter size: {3 x 3}  Size of max-pooling: {(2, 2)}  Optimizer: {Adam}  Activation function: {combination of activation function}  Epochs: {2000}  Batch size: {200}  Dropout rate: {0.5,0.25}  Number of layers: 3  This information has been added to the revised version of the paper (Table 4). |
| The image dataset generation process needs to be discussed in detail. | The process of creating an image is as follows.  1. Daily data for 5 years (2015-2019) was collected on 789 companies included in the KOSPI Index calculation in the Dataguide program.  2. Images were composed of data collected on a monthly basis.  3. According to the image characteristics shown in Table 2, 30 images of various types were created for each month with RStudio software.  4. After the images were created, a label for each was automatically generated with the name of the image, which was then compared with the closing price of the stock price 5 days later. Please see the following example.    5. Finally, if the image name rate of return was greater than 0, 1 was automatically added to the end of the label; otherwise, 0 was automatically added.  This text has been added to section 4.1. |
| There are many grammatical and syntactic errors inside. A native speaker can fix it. | Thank you. The paper was edited by a native speaker who is familiar with technical papers. |

Reviewer 2

| Comments | Response |
| --- | --- |
| Scope of the manuscript is limited. The research work uses only image features for price prediction. | The study of predicting stock prices with images has begun. Many example studies have been cited in the paper. However, there is still no research on how the characteristics of a chart affect the performance of deep learning-based stock price predictions. This is the novelty of this paper.  The results (Table 8) suggest that using chart images is more useful for stock price prediction than numeric and text-based methods.  Also, the authors are considering a method of combining image-based prediction and technical indicator-based prediction in future research. This information has been newly added in the discussion section. |
| The manuscript uses only CNN on the image data for prediction. There are state-of-the-art methods that should also be used and compare with CNN. | We agree with your statement. In the current study, only the CNN algorithm is used, but in future work, we will include algorithms such as BI-GRU and LSTM among RNNs. For the purposes of this paper, the main focus is differences in the performance of deep learning algorithms according to image characteristics. Our results inspire us to further research in this area.  This information has been added to the section discussing the limitations of this paper. |
| The main idea of this research work is to use image features for prediction. But there is not enough information, how the images and there features are generated. So in section 4.1, please provide some more information how the chart images were created. | The process of creating an image is as follows.  1. Daily data for 5 years (2015-2019) was collected on 789 companies included in the KOSPI Index calculation in the Dataguide program.  2. Images were composed of data collected on a monthly basis.  3. According to the image characteristics shown in Table 2, 30 images of various types were created for each month with RStudio software.  4. After the images were created, a label for each was automatically generated with the name of the image, which was then compared with the closing stock price 5 days later. Please see the following example.    5. Finally, if the image name rate of return was greater than 0, 1 was automatically added to the end of the label; otherwise, 0 was automatically added.  This text has been added in section 4.1. |
