# Supplementary material for: Impact of chart image characteristics on stock price prediction with a convolutional neural network
Source: PLoS One. 2021 Jun 23;16(6):e0253121. doi: 10.1371/journal.pone.0253121 (PMC8221485; doi:10.1371/journal.pone.0253121)
Supplement: Attachment — Submitted filename: Revision Summary2021.05.14.docx [file pone.0253121.s002.docx]

**Revision Summary**

ID: PONE-D-20-29152.R2

Paper Title: Impact of Chart Image Characteristics on Stock Price Prediction with a Convolutional Neural Network

Reviewer 1

| Comments | Response |
| --- | --- |
| • The authors still didn't provide sufficient detail on model training and ablation study. It will be worthy for publication after such minor revision. | • Thank you for your comments. Details on model training have been added in Table 4 (activation function, learning rate, number of layers). |

Reviewer 2

| Comments | Response |
| --- | --- |
| • The objective of the research should be clearly defined in the last paragraph of the introduction section.  • Add the advantages of the proposed system in one quoted line for justifying the proposed approach in the Introduction section. | • The objective of the research has been added in the last paragraph of the introduction.  • The advantages of the proposed system have been outlined in the last paragraph of the introduction. |
| • The motivation for the present research would be clearer, by providing a more direct link between the importance of choosing your own method.  The authors can cite the following references  Analysis of dimensionality reduction techniques on big data  Deep neural networks to predict diabetic retinopathy  Antlion re-sampling based deep neural network model for classification of imbalanced multimodal stroke dataset | • The motivation for our research has been described at the end of the introduction section.  • The following sentences have been added in the revised version of the manuscript:  In fact, performing the CNN algorithm using image datasets or multimodal datasets has been actively attempted in various domains such as medicine (Reddy et al., 2020b). In addition, prediction performance has been improved by selecting an appropriate preprocessing method according to the domain (Gadekallu et al., 2020; Reddy et al., 2020a). However, only a few studies have mentioned a preprocessing method that selects the optimal image characteristics in advance for stock price prediction.  • Correspondingly, we also added the following references:  Reddy, G. T., Reddy, M. P. K., Lakshmanna, K., Kaluri, R., Rajput, D. S., Srivastava, G., & Baker, T. (2020a). Analysis of dimensionality reduction techniques on big data. *IEEE Access*, *8*, 54776-54788.  Gadekallu, T. R., Khare, N., Bhattacharya, S., Singh, S., Maddikunta, P. K. R., & Srivastava, G. (2020). Deep neural networks to predict diabetic retinopathy. *Journal Of Ambient Intelligence and Humanized Computing*, 1-14.  Reddy, T., Bhattacharya, S., Maddikunta, P. K. R., Hakak, S., Khan, W. Z., Bashir, A. K., ... & Tariq, U. (2020b). Antlion re-sampling based deep neural network model for classification of imbalanced multimodal stroke dataset. *Multimedia Tools and Applications*, 1-25. |
