# Supplementary material for: Impact of chart image characteristics on stock price prediction with a convolutional neural network
Source: PLoS One. 2021 Jun 23;16(6):e0253121. doi: 10.1371/journal.pone.0253121 (PMC8221485; doi:10.1371/journal.pone.0253121)
Supplement: Attachment — Submitted filename: Revision Summary2021.05.29.docx [file pone.0253121.s003.docx]

**Revision Summary**

ID: PONE-D-20-29152.R3

Paper Title: Impact of Chart Image Characteristics on Stock Price Prediction with a Convolutional Neural Network

Editor

| Comments | Response |
| --- | --- |
| • They claimed that they have addressed all the comments of the reviewers in the response sheet but in the manuscript they are not reflected. For instance in teh response sheet the authors claimed that:  Correspondingly, we also added the following references: Reddy, G. T., Reddy, M. P. K., Lakshmanna, K., Kaluri, R., Rajput, D. S., Srivastava, G., & Baker, T. (2020a). Analysis of dimensionality reduction techniques on big data. IEEE Access, 8, 54776-54788. Gadekallu, T. R., Khare, N., Bhattacharya, S., Singh, S., Maddikunta, P. K. R., & Srivastava, G. (2020). Deep neural networks to predict diabetic retinopathy. Journal Of Ambient Intelligence and Humanized Computing, 1-14. Reddy, T., Bhattacharya, S., Maddikunta, P. K. R., Hakak, S., Khan, W. Z., Bashir, A. K., ... & Tariq, U. (2020b). Antlion re-sampling based deep neural network model for classification of imbalanced multimodal stroke dataset. Multimedia Tools and Applications, 1-25  But this is not reflected in the paper. | Thank you for your careful review. We added missing references and modified the bibliography form to fit PLOS-ONE. All other comments are already reflected from the R2 version of the paper. |
